# Hypoxia-Modified Cancer Cell Metabolism

**DOI:** 10.3389/fcell.2019.00004

**Published:** 2019-01-29

**Authors:** Wafaa Al Tameemi, Tina P. Dale, Rakad M. Kh Al-Jumaily, Nicholas R. Forsyth

**Affiliations:** ^1^Faculty of Medicine and Health Sciences, Institute for Science and Technology in Medicine, Keele University, Staffordshire, United Kingdom; ^2^Department of Biology, College of Science, University of Baghdad, Baghdad, Iraq

**Keywords:** hypoxia, metabolism, Warburg effect, HIF, Glut-1, glycolysis, mitochondria

## Abstract

While oxygen is critical to the continued existence of complex organisms, extreme levels of oxygen within a system, known as hypoxia (low levels of oxygen) and hyperoxia (excessive levels of oxygen), potentially promote stress within a defined biological environment. The consequences of tissue hypoxia, a result of a defective oxygen supply, vary in response to the gravity, extent and environment of the malfunction. Persistent pathological hypoxia is incompatible with normal biological functions, and as a result, multicellular organisms have been compelled to develop both organism-wide and cellular-level hypoxia solutions. Both direct, including oxidative phosphorylation down-regulation and inhibition of fatty-acid desaturation, and indirect processes, including altered hypoxia-sensitive transcription factor expression, facilitate the metabolic modifications that occur in response to hypoxia. Due to the dysfunctional vasculature associated with large areas of some cancers, sections of these tumors continue to develop in hypoxic environments. Crucial to drug development, a robust understanding of the significance of these metabolism changes will facilitate our understanding of cancer cell survival. This review defines our current knowledge base of several of the hypoxia-instigated modifications in cancer cell metabolism and exemplifies the correlation between metabolic change and its support of the hypoxic-adapted malignancy.

## Introduction

Hypoxic regions, areas of reduced tissue oxygen levels, are found in many solid tumors occurring as a consequence of the disordered vasculature developed to supply oxygen to the rapidly growing tumor. In cancer patients tumor hypoxia leads to a poor prognosis (Vaupel, 2008) due to the potential of increased malignancy, resistance to both chemotherapy and radiation treatment, and an increased likelihood of metastasis (Höckel and Vaupel, 2001). In comparison to normal human tissues where the oxygen tension typically exceeds 40 mmHg, in tumors an oxygen tension of 0–20 mmHg may persist (Vaupel et al., 1989). Hypoxia typically arises in solid tumors at a distance of approximately 100 μm from a functional blood vessel (Helmlinger et al., 1997).

**FIGURE 1:**
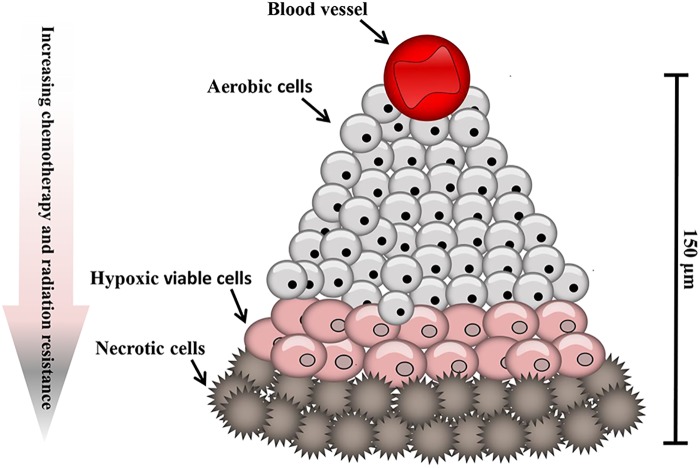
Hypoxic regions of solid tumors. Tumors contain regions of oxygenated cells situated near to blood vessels, becoming increasingly hypoxic with increased distance from a functional blood supply.

Tumor cells are exposed to a continuum of oxygen concentrations and consequently solid tumors are comprised of three tissue regions: the normoxic, hypoxic and necrotic (Figure 1). Situated nearby functional blood vessels normoxic cells are typically viable and proliferative. At distances of 150 μm from patent blood vessels cells may become anoxic, giving rise to patches of necrosis. Peri-necrotic cells are typically hypoxic and capable of existing at very low oxygen concentrations (PO_2_ ≤ 1%) (Hall and Giaccia, 2006). In normal cells, hypoxia typically leads to cell death. Counter-intuitively, however, hypoxia can induce genomic changes that enable tumor cells to adapt to poor nutrition and the hostile microenvironment, thus remaining viable. Consequently, hypoxia exerts a selection pressure that leads to the survival of subpopulations of viable cells with the genetic machinery for malignant progression (Vaupel and Harrison, 2004). Tumors can overcome the proliferation limitations posed by the stressful microenvironment by stimulating the production of new blood vessels *via* the release of hypoxia-inducible angiogenic factors, such as vascular endothelial growth factor (VEGF) to develop a new blood supply (Seo et al., 2014). Paradoxically, following neovascularization in solid tumor tissue, which consists of poorly organized, elongated, dilated, twisted and blind-ended blood vessels, oxygen supply for the tumor may still be deficient (Helmlinger et al., 1997).

In human tumors hypoxia presents as one of two classes, chronic or acute forms, based on the conventional categorization common in research and medical oncology (Höckel and Vaupel, 2001; Vaupel et al., 2001). As noted by Vaupel et al. (2001) and Vaupel and Harrison (2004), the structural and functional abnormalities arising from the chaotic vasculature and structure of a tumor, including dilated, elongated and twisted blood vessels, poor endothelium, reduced functional cell receptors, no regulation of blood flow, which results in spontaneous stasis, result in poor oxygen delivery as a consequence of inadequate blood flow. This form of hypoxia is termed ischemic hypoxia and is generally transient (Vaupel et al., 2001; Vaupel and Harrison, 2004). Chronic, diffusion-limited hypoxia results from an imbalance of oxygen supply and demand due to an increase in diffusion distance with tumor growth, where rapid tumor expansion results in tumor tissue at distances further than 70–150 μm from patent blood vessels receiving insufficient oxygen. Occasionally, anemic hypoxia can arise, initiated by a decreased capacity of the blood to transport oxygen when anemia is induced by chemotherapy (Vaupel et al., 2001). Both acute and chronic hypoxia are correlated with poor patient outcome and an aggressive tumor phenotype (Williams et al., 2001; Vaupel and Harrison, 2004).

## Cellular Responses and Adaptations to Hypoxia

**FIGURE 2:**
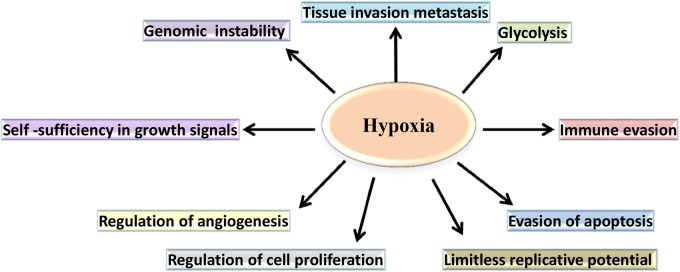
The role of hypoxia in the cancer-specific biological pathways. In hypoxic conditions, cancer cell metabolism undergoes a shift from oxidative phosphorylation to aerobic glycolysis. Additionally, hypoxia regulates cell proliferation and supports evasion of apoptosis by the tumor cells. Furthermore, hypoxia contributes to the changes that confer limitless replicative potential and to the expression of genes, allowing invasion, and metastasis.

Hypoxia induces both proteomic and genomic changes within tumor cells (Figure 2). Proteomic changes may initiate cell cycle arrest, differentiation, necrosis and apoptosis (Lee and Lin, 2017). Additionally, hypoxia- induced proteomic changes may also stimulate tumor growth, invasion and metastasis by facilitating acclimatization and survival in a hostile, nutrient-deprived environment (Vaupel and Harrison, 2004). At a molecular level, the adaptation of tumor cells to hypoxic stress is regulated largely by hypoxia-inducible factor (HIF), a transcription factor which accumulates in response to decreased cellular oxygen levels (Schito and Rey, 2017; Wolff et al., 2017).

## Hypoxia-Inducible Factors (HIFs) the Key Operator in Hypoxia Signaling

**FIGURE 3:**
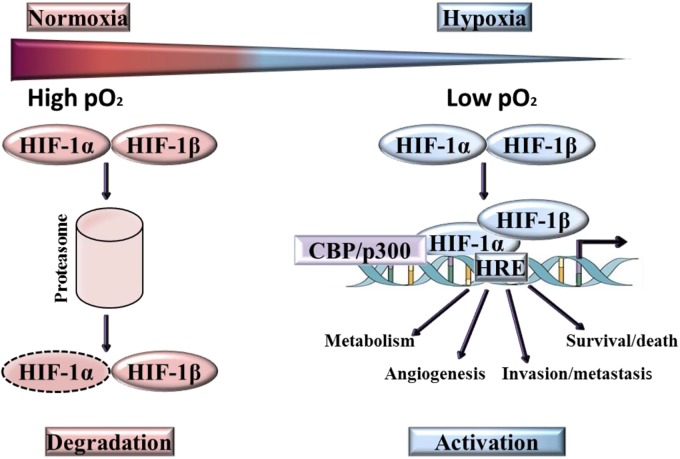
Activation and degradation of the hypoxia inducible factor-1α (HIF-1α). In normoxia HIF-1α is rapidly degraded, while it accumulates in hypoxic conditions. HIF-1α associates with HIF-1β and the resulting heterodimer binds to the hypoxia response element (HRE) of target genes.

Three human HIF family members have been identified, HIF-1, HIF-2, and HIF-3, these heterodimers comprise of α subunit and β subunit, which dissociate in normoxic conditions (Figure 3). Of the three isoforms, HIF-1 is frequently overexpressed in tumor cells (Weidemann and Johnson, 2008; Robinson et al., 2017), with HIF-2 [endothelial PAS domain protein 1 (EPAS1)] strongly expressed by subsets of tumor-associated macrophages (Talks et al., 2000) and HIF-3 expressed in pulmonary alveolar epithelial cells (Li et al., 2006) and in human kidney (Yang et al., 2015). The most extensive characterization has been provided for HIF-1α. Although the DNA binding and dimerization domains of HIF-1α and HIF-2α are similar from a structural perspective, the transactivation domains of these two forms are dissimilar. This may be the reason for the genome wide screen detection of the binding of both forms to identical (hypoxia-response elements) HRE consensus sites, but with a different transcriptional responses being activated (Mole et al., 2009). Another difference is that HIF-1α has all-pervasive expression, whereas HIF-2α expression is more limited to specific tissues (Hu et al., 2003; Bhatia et al., 2013). On the whole, the two forms react to hypoxia through different biological actions (Loboda et al., 2010). To give an example, the transcription of genes that encode enzymes participating in glycolysis is regulated solely by HIF-1α as observed by Hu et al. (2003). By contrast, HIF-2α is believed to play a role in adjustment to high altitudes (van Patot and Gassmann, 2011). In the case of human colon cancer, HIF-1α staining was strong whilst HIF-2α staining was weak in late stage tumors, whereas the opposite has been documented in early stage tumors. This phenomenon has prompted Imamura et al. (2009) to deduce that the involvement of HIF-1α and HIF-2α in human colon cancer was not the same (Imamura et al., 2009). On the other hand, the transactivation domain is absent from HIF-3α which means that this form possesses a suppressive effect, preventing HIF-1α from initiating transcription by binding to it. Due to this action, HIF-3α is also referred to as the ‘inhibitory Per-Arnt-Sim PAS domain’ (IPAS) (Weidemann and Johnson, 2008).

The transcription of HIF-1α does not require oxygen, however, in normoxia, HIF-1α is rapidly degraded (Kaelin et al., 2016). Degradation of HIF-1α under normoxic conditions is a ubiquitin-mediated process, activated by the tumor suppressor protein pVHL (von-Hippel-Lindau protein). In contrast, ARNT ((Aryl Hydrocarbon Receptor Nuclear Translocator), also known as HIF-1β) is not downregulated by oxygen and is constitutively present. At low oxygen levels, HIF-1α is stabilized and translocates from the cytoplasm into the nucleus where it binds to its heterodimeric partner ARNT, inducing a conformational change and the formation of active HIF-1 (Zimna and Kurpisz, 2015). Over 100 genes are regulated directly and indirectly by HIF (Goda and Kanai, 2012; Semenza, 2012a), the active form of which binds to a HRE in the target gene promoter region. These genes are implicated in a myriad of functions, including the promotion of angiogenesis, erythropoiesis, cell growth and proliferation, invasion/metastasis and metabolic adaptation. Many of the genes induced by HIF-1 are highly expressed in tumors relative to normal tissues, for example VEGF, Glucose transporter 1, Glucose transporter 3, Insulin-like growth factor binding protein-1 and -3, Insulin-like growth factor II, Transforming growth factor-β3 and p21 (Maxwell et al., 2001; Masoud and Li, 2015). In HIF induced angiogenesis, the extent of neo-vascularization implicates HIF-mediated expression of the two key angiogenic factors, VEGF-A and angiopoietin-2 (Brahimi-Horn and Pouysségur, 2007).

## The Role of Hypoxia in Cancer Stem Cells (CSCs)

In common with normal adult stem cells (ASCs), cancer stem cells (CSCs) are undifferentiated self-renewing cells able to form a heterogeneous population of daughter cells. Under atmospheric oxygen conditions (21%), *in vitro* stem cells lose their stemness characteristics (Simon and Keith, 2008). *In vivo*, ASCs are maintained in hypoxic conditions, typically defined as 1% oxygen; an example being haematopoietic stem cells (HSCs) that reside in the hypoxic endosteal regions of bone marrow (Kubota et al., 2008). CSCs also favor hypoxic sites within tumors (Das et al., 2008), enabling the cells to maintain their undifferentiated state. As with ASCs, differentiation occurs in *in vitro* populations of cells under physiologically normal levels of oxygen (Jögi et al., 2002; Heddleston et al., 2009).

The stimulation of cancer cell stemness by HIFs is well documented, with studies revealing HIF-1α and HIF-2α to be central to CSC stemness. Upregulation of HIF-1α, which heralds CSC proliferation, is essential for CSC survival in hypoxic conditions (Lau et al., 2017). HIF-2α is also key to CSC survival and it co–localizes with CSC markers. HIF-1α and HIF-2α are both essential for maintaining stem–like properties and CSC survival (Li et al., 2009). The contributions made by HIF-1α and HIF-2α in maintaining the stem–like state of CSCs differ (Li et al., 2009). As previously mentioned HIF-1α binds to and stimulates the genes for survival in low oxygen conditions, such as those of the glycolysis pathway. On the other hand, the target structures of HIF-2α are stem cell upkeep genes, including *Notch*, octamer-binding transcription factor 4 (*Oct4*) and the sex determining region Y-box 2 (*Sox2*). This differentiation of function suggests that stemness may be influenced more by HIF-2α than HIF-1α (Covello et al., 2006; Keith et al., 2012).

A significant challenge for clinicians treating cancer is chemotherapy resistance leading to a poor therapeutic response, and in turn, to relapse (Dean et al., 2005). One mechanism of resistance can be attributed to the capacity of CSCs to evade and resist chemotherapy by virtue of their stem cell qualities. CSCs can adopt slow cycling growth, known as quiescence, enabling the cells to avoid those therapies that target rapidly dividing cells; additionally, the survival signaling pathways can be modified preventing cell death (Clarke and Hass, 2006). Moreover, the self-renewing capability of CSCs leads them to act as reservoir cells, facilitating cancer reoccurrence (Clarke and Hass, 2006; Holohan et al., 2013; Schöning et al., 2017). Using a murine model Saito et al. (2010) used chemical stimuli to induce CSCs present in the hypoxic endosteal region of the bone marrow to enter the cell cycle. When combined with chemotherapy, the survival of mice with leukemia was increased by this treatment methodology (Saito et al., 2010). Thus, maintaining CSCs in hypoxic microenvironments sustains the cells’ quiescent state and consequently, they are able to resist most chemotherapy interventions (Schöning et al., 2017).

## The Role of Hypoxia in the Tumor Stroma

Rather than only being determined by cancer cells, cancer progression is the product of the entire tumor microenvironment, including cancer cells, extracellular matrix (ECM) and stroma (Hanahan and Coussens, 2012). Through inhibition of the expression of Bid, the pro-apoptotic Bcl-2-family protein, HIF-1 reduces apoptosis (Erler et al., 2004). HIF-1 also stimulates expression of survivin, an apoptosis inhibitor (Peng et al., 2006). These mechanisms enable cancer cells to endure; they are protected from the punishing hypoxic environment and reduce the cells’ susceptibility to drug actions (Erler et al., 2004).

The growth of cancer cells can result from autocrine signaling. By having growth factor receptors on the membrane, these cells can stimulate their own growth and survival by secreting growth factors. During tumor development stromal cells are in close proximity with cancer cells thus these same growth factors modify the phenotypic behavior of surrounding stromal cells to promote tumors (Semenza, 2012b). Furthermore, cancer cell invasion and metastasis is promoted by hypoxia. Under such conditions, cancer cells experience greater motility and invasiveness by encouraging epithelial to mesenchymal transition. The mechanism responsible for this is upregulated expression of the transcription factors Snail Family Transcriptional Repressor 1 (Snail1), Snail Family Transcriptional Repressor 2 (Snail2), and Twist. As a result, the expression of *E*-cadherin, which is a key component of adherens junctions, is downregulated (Schwab et al., 2012). Cancer-associated fibroblasts (CAFs) are primary tumor stroma components (Cirri and Chiarugi, 2012; Lau et al., 2017). CAFs act in a paracrine manner, encouraging tumor growth and sustaining CSCs’ stemness property (Forsberg et al., 1993; Giannoni et al., 2010). Research shows that CAFs are a heterogeneous population, of which the majority adopt the activated phenotype, exhibiting increased secretion of ECM, growth factors and proteases, greater contractile force, and proliferation activity (Tao et al., 2017). There are numerous origins of CAFs, varying with different forms of cancer. Several studies reveal that by secreting signaling molecules, cancer cells cause resting fibroblasts to become CAFs. Such signals include basic fibroblast growth factor (bFGF), interleukin-6 (IL-6), platelet-derived growth factor (PDGF) and transforming growth factor beta (TGF-*β*) (Tao et al., 2017).

HIF-1 contributes to the behavior of normal fibroblasts and CAFs; furthermore, HIF-1α demonstrates tumor-promoting capability. Elevated tumor growth has been observed in studies that use the active form of HIF-1α expressed by human skin fibroblasts co-injected with MDA-MB-231 cells. The same effect was not detected with HIF-2α. It is proposed that activating HIF-1α initiates autophagy and aerobic glycolysis; this provides cells surrounding the cancer cells with the energy necessary to promote their growth (Chiavarina et al., 2010).

## HIF Hydroxylation

Under conditions of normoxia, hydroxylation of the two proline residues located in the oxygen dependent degradation domain (ODDD) region by prolyl-4-hydroxylase domain (PHD) enzyme and lysine (Lys532) acylation by acetyl-transferase-arrest-defective-1 (ARD-33 1) enzyme activity (Dengler et al., 2014) destabilizes the HIF-α subunits. Subsequent to hydroxylation and acetylation, HIF-α is identified by p-VHL tumor suppressor resulting in poly-ubiquitination by E3 ubiquitin-ligase; and this complex is then further degraded by proteasome 26S (Masoud and Li, 2015). A further hydroxylation reaction takes place on Asparagine 803 on the C-terminal domain of HIF-α. This hydroxylation is due to factor inhibiting HIF (FIH), which restricts co-activation with p300, thereby inhibiting HIF transcriptional activation (Masoud and Li, 2015). Prolyl-hydroxylase and asparagine hydroxylase are both members of the oxygen-dependent hydroxylase families whose catalytic properties are dependent upon oxygen, and are viewed as the cellular detectors for oxygen concentration (Schofield and Ratcliffe, 2004). Under hypoxic conditions, the absence of oxygen, which constitutes the substrate for PHDs and FIH, results in a cessation of the hydroxylation process, preventing the binding of HIF-α to the p-VHL (Arsenault et al., 2015; Maxwell et al., 2017). This leads to the build-up of HIF-1α in its stable isomer, translocation across the nuclear membrane and binding to the β- subunit to generate the active HIF dimer. This subsequently binds to co-activators, e.g., CREB-binding protein CBP/p300, transcription intermediary factor 2 and steroid–receptor activator activators to up-regulate gene transcription (Ke and Costa, 2006; Luo et al., 2011).

Prolyl-4-hydroxylase domain enzyme activity represents the connection between oxygen sensing and energy generation by the Kreb’s Cycle. Secondary metabolites of the Kreb’s Cycle are key to the PHDs activity, whilst end-products of the Kreb’s Cycle (e.g., succinate and fumarate) inhibit PHD enzyme activity (Isaacs et al., 2005; Koivunen et al., 2007). Mutations involving metabolic genes [e.g., fumarate hydratase (FH), the subunits of succinate dehydrogenase (SDH), and isocitrate dehydrogenase (IDH1 and IDH2)] can give rise to a condition termed pseudo-hypoxia by formation of a reduced variety of 2-oxoglutarate, (R)-2-hydroxyglutarate, as an oncometabolite, involving activation of HIF-1, irrespective of a normal oxygen environment (Chowdhury et al., 2011; Koivunen et al., 2012).

At least four isoforms of the prolyl hydroxylase domain enzyme (PHD1-4) have been identified so far in mammalian tissues. The PHDs all possess a double strand β-helix core fold, which are also termed jelly roll folds (Karuppagounder and Ratan, 2012). All PHD isoforms are able to hydroxylate the LXXLAP motif in HIF-α subunits and this sequence is also present in other molecules, including the nuclear factor of kappa light polypeptide gene enhancer in B-cells inhibitor (IκBα), RNA polymerase and β-adrenergic receptors (Fong and Takeda, 2008). The HIF mechanism is halted under conditions of normoxia by the inhibitory influence of PHD, whilst under conditions of hypoxia; PHDs are inactive, resulting in an active HIF pathway (Fong and Takeda, 2008). PHDs are viewed as the cellular oxygen sensor, because they express a high affinity to oxygen, possessing a Km-value within the 230–250 μM range, when compared with alternative hydroxylase enzymes, such as collagen hydroxylase (Olcina et al., 2018). So as to ensure optimum activity, PHDs have a requirement for oxygen molecules: one oxygen atom combines with proline to produce hydroxyl-proline and the other oxygen atom is utilized for de-carboxylation of 2-OG to produce succinate (Gerald et al., 2004). Furthermore, the PHDs also need non-haem-Fe^2+^ in order to engage in catalytic activity; which binds to the His-X-Asp/Glu-Xn-His motif to promote such enzymatic activity. In addition, the PHD also requires ascorbic acid to recycle Fe^2+^ to its active form (Hancock et al., 2015). The activity of PHDs is inhibited by hypoxia, nitric oxide, ROS, 2-OG analogs and cobalt chloride (Maxwell et al., 2017).

Prolyl-4-hydroxylase domains can be inhibited through multiple methods under normoxic conditions: via disruption of the Fe^2+^ equilibrium by application of an iron chelator such as deferoxamine (DFO) or via application of a competitive inhibitor, such as cobalt chloride (Deppe et al., 2016). These non-specific chemicals result in the potential for off-target effects to be significant (Deppe et al., 2016). An alternative approach for modulation of PHDs activity is via application of 2-OG analogs, including: L-Mimosine (L-Mim); dimethyloxalyl-glycine (DMOG); 3,4-dihydroxybenzoate (3,4-DHB); and *N*-[[1,2-dihydro-4-hydroxy-2-oxo-1-(phenylmethyl)-3-quinolinyl] carbonyl]-glycine (IOX2). IOX2 possesses significantly greater potency than DMOG, where relative IC50 values are 0.022 μM and 5 μM respectively. IOX2 is able to inhibit both PHD-2 and FIH with greater than 5000 times the selectivity for PHD-2 than histone demethylases (Chowdhury et al., 2013).

## Epigenetic Interactions With the Metabolism of Tumor Cells

Gene expression is dramatically influenced by metabolic changes which are associated with cancer. Whilst metabolite profiles may not have much influence at the genetic level, it would seem that they do exert a significant influence on epigenetic regulation of gene expression. Epigenetic factors can be defined as heritable factors, which in this case influence gene expression, without requiring modification of DNA sequences (Wong et al., 2017). Such epigenetic regulation of gene expression can be extremely malleable in response to a wide variety of environmental parameters (Herceg and Vaissière, 2011; Feil and Fraga, 2012). Epigenetics fundamentally involves the chemical alteration of DNA and histone molecules and constitutes a mechanism which inherently connects nutritional state with gene expression. Consequently, it is possible for metabolic changes to override the epigenomic apparatus within cancer cells (Gupta et al., 2014; Johnson et al., 2015). Similarly, epigenetic deregulation in cancer at least partly facilitates altered gene expression (Wong et al., 2017).

Epigenetics and metabolism in cancer are connected by four interlinking pathways: (1) metabolic rewiring may influence the required cofactor availability for epigenetic enzyme alteration; (2) oncometabolites functioning as agonists and/or antagonists for such epigenetic enzyme alteration may be generated, influencing the epigenetic environment; (3) conversely, epigenetic dysfunction can directly alter metabolism by influencing metabolic enzyme expression; (4) the signal transduction cascades contributing to the regulation of cellular metabolism may be altered (Wong et al., 2017). The role played by epigenetics in cellular responses to hypoxia is becoming widely acknowledged. This type of epigenetic control may function in tandem with the HIFs or, may provide a robust pathway for the maintenance of a hypoxia-adapted cellular phenotype for some time following the HIF initiation of immediate response mechanisms. This includes: the part played by epigenetics in both the stabilization and bonding of HIF to its transcriptional targets; the part played by histone demethylase enzymes subsequent to direct HIF transactivation; and, the effect of a hypoxic mileau on global histone modification responses and DNA methylation (Watson et al., 2010). There is growing support for the suggestion that HIF activity is superimposed over a background of epigenetic alterations which are a prerequisite for assessing any hypoxic response. In particular, epigenetic alterations, at both the level of the DNA and histones proffer the capability to specify HIF binding to target gene promoters. Furthermore, hypoxia is inherently a potent inducer of chromatin remodeling by regulating enzymes responsible for DNA methylation modulation and histone alterations (Watson et al., 2010). It is also critical to observe that chronic hypoxia can lead to alterations in gene expression which remain independent of the classical HIF mechanism. This can take place via changes in the methylation status of gene sequences or, changes to the normoxic histone code, potentially via prolonged modifications to epigenetic altering enzymes (Watson et al., 2010; Vanharanta et al., 2013). At the moment, there are four different views on the relationship between epigenetics and hypoxia: (1) HIF stabilization is affected by the epigenetic control of VHL and PHD3 expression; (2) epigenetic pathways control HIF binding by ensuring a transcriptionally active chromatin configuration in and in the vicinity of HIF binding sites (this may be due to the HIF-1α coactivation complex or to direct changes to the HRE binding sites, thereby preventing HIF binding); (3) a major proportion of histone demethylase enzymes are direct HIF-1 target genes which consequently play a major part in the control of transcription during responses to hypoxia; (4) major wide-reaching changes in histone alterations and DNA methylation take place following exposure to hypoxia (Watson et al., 2010; Wong et al., 2017).

## The Influence of Epigenetics in HIF-α Stabilization Control

Susceptibility to VHL-dependent ubiquitination and degradation is conferred by PHD-dependent hydroxylation of HIF-α under aerobic conditions (Hatzimichael et al., 2009). VHL tumor suppressor gene mutations in humans are known to result in a predisposition to a range of tumors, such as renal cell carcinoma, hemangioblastoma of the central nervous system, and phaeochromocytoma. Furthermore, VHL loss of function mutations have been extensively investigated revealing that expression of VHL can be blocked by promoter hypermethylation in renal cell carcinoma and multiple myeloma (Hatzimichael et al., 2009). Loss of VHL expression, as a consequence of promoter hypermethylation, potentially represents a key epigenetic mechanism which can lead to exacerbation of HIF activity (Hatzimichael et al., 2009, 2010). The original suggestion that the hypoxic response is largely dependent upon the cooperation of epigenetics was proposed following examination of the HIF-1α coactivation complex, where a number of epigenetic modifying enzymes were identified in direct contact with HIF-1α during the initial cellular hypoxic response. It is known that the histone acetyltransferase enzyme CBP/p300 directly associates with HIF-1α and takes part in the coactivation of a network of hypoxia-inducible genes (Kallio et al., 1998; Watson et al., 2010). Whilst many of the current perspectives on the relationship between hypoxic response and epigenetic mechanisms concentrate on the known interactions with HIF-1α, it is considered that epigenetics is likely to have additional important roles relating to cell adaptation and survival under chronic hypoxic conditions subsequent to the initial HIF-1α-induced cellular response (Watson et al., 2010; Vanharanta et al., 2013; Hancock et al., 2015).

## Changes in Metabolism That Are Conducive to Redox Status

Every cell produces reactive oxygen species (ROS), a varied group of radical species constituting a normal by-product of metabolic activities. The attributes and downstream effects of ROS vary according to the levels in which these radical species occur. When their concentration is low, ROS subject kinases and phosphatases to post-translational alterations, thus stimulating cells to proliferate and survive (Giannoni et al., 2005; Cao et al., 2009). NADPH and NADPH oxidase (NOX) are usually responsible for production of ROS in low concentration, which is necessary for signaling events associated with homeostasis. When their concentration is moderate, ROS trigger the expression of stress-responsive genes (e.g., HIF-1α) (Gao et al., 2007; Chandel, 2010).

A notable incongruity is the fact that stabilization and activation of HIF-1 are associated not only with conditions of reduced levels of oxygen, but also with ROS. Guzy et al. (2005) reported that under normoxia HIF-1α was stabilized and the expression of HRE-reporter constructs was increased when hydrogen peroxide was added. Furthermore, under the same conditions of normoxia coupled with addition of hydrogen peroxide, HRE-luciferase reporter activity can be displayed by Hep3B cells, which lack mitochondrial electron transport function (Chandel et al., 2000). Meanwhile in a different study, breast tumor cells from mouse models were exposed to nitric oxide as a way of demonstrating the molecular basis of HIF-1 stabilization by ROS. Under normoxia, a particular cysteine residue in the Oxygen-dependent degradation ODD domain of HIF-1α underwent nitrosylation when nitric oxide was added. In this way, the binding of the von Hippel-Lindau (VHL) protein to HIF-1α was not possible and as a result, HIF-1α did not deteriorate (Weidemann and Johnson, 2008). Involvement in the HIF-1 signaling pathway under conditions of oxygen deficiency (hypoxia) is an additional function that is thought to be undertaken by ROS. As has been reported by several researchers, e.g. (Guzy et al., 2005; Mansfield et al., 2005) the cells that did not have functional mitochondria, and implicitly had low levels of ROS, failed to achieve stabilization of HIF-α under circumstances of hypoxia. Furthermore, it was observed that HRE-luciferase reporter activity was diminished when excessive expression of catalase in human 293 cells suppressed hydrogen peroxide under hypoxia; what was implied from this was that HIF-1α activity was reduced, and therefore hydrogen peroxide was added in order to recover it. This may be related to fact that HIF participates in ROS regulation while ROS play a role in HIF expression and activity (Kotake-Nara and Saida, 2007; Owusu-Ansah et al., 2008; Zeng et al., 2011). The conclusion that has been derived from such observations was that, under conditions of hypoxia, HIF-1α could only be stabilized if hydrogen peroxide was present in the cytosol (Chandel et al., 2000).

Earlier research has demonstrated that ROS inactivate PHDs via oxidation of the ferrous ion which is required for their catalytic mechanism, thereby stabilizing HIF-1α (Yang et al., 2014). Vitamin C has been demonstrated to reduce HIF-1 levels by blocking the oxidation of the catalytic ferrous ion (Gao et al., 2007; Calvani et al., 2012). Furthermore, it has also been recently reported that the anti-carcinogenic influence of antioxidants, such as *N*-acetyl cysteine (NAC) and vitamin C, are without doubt HIF-1-dependent in murine simulations of Myc-mediated tumor genesis (Calvani et al., 2012). Earlier research has also demonstrated that under conditions of hypoxia, melanoma cells are subject to constant oxidative stress as a consequence of raised intracellular levels of ROS, resulting from mitochondrial complex III deregulation (Comito et al., 2011). Mitochondrial ROS have been found to play a predominant role in ROS formation and subsequent HIF-1 stabilization in hypoxic environments (Bell et al., 2007), as well as under non-hypoxic situations (Patten et al., 2010). In addition to mitochondria, NADPH oxidases have also been identified as playing a significant part in ROS generation and in redox-dependent HIF-1 stabilization (predominantly under normoxic conditions) (Lee et al., 2011). Calvani et al. (2012) demonstrated that production of hypoxic ROS is a primary role for HIF-1 stabilization, and activation of its transcriptional response (Calvani et al., 2012). This was demonstrated by showing how hypoxia evokes a redox-dependent stabilization of HIF-1 in rat phaeochromocytoma PC12 cells, which subsequently depends upon NADPH oxidase-driven ROS, Ca^2+^ and the mammalian objective of rapamycin (mTOR) signaling (Calvani et al., 2012). Enhancing ROS production under conditions of hypoxia may initiate a redox adaptation response which allows cancer cells to survive, as a consequence of raised tolerance to exogenous stress, up-regulation of survival molecules and enhanced drug inactivation capabilities (Pagé et al., 2008).

## The Role of Hypoxia-Regulated MicroRnas in Cancer Metabolism

MicroRNAs (miRNAs) are small, endogenous RNA molecules; they measure 18–24 nucleotides in length, and occur in eukaryotes only. They do not code proteins, but regulate post-transcriptional and translational gene expression. These molecules have multiple actions contributing to diverse normal and pathological cellular processes, including cell death, development, metabolism, neuronal patterning and oncogenesis (Pocock, 2011; Yu et al., 2012; Agrawal et al., 2016). Research indicates that a group of microRNAs are regulated by HIF-1, and in other instances, HIF-1 is the target of microRNAs. Many aspects of tumorigenesis are attributed to the interaction between microRNAs and HIF-1; such aspects include angiogenesis, apoptosis, cell cycle regulation, metabolism, metastasis, proliferation and anticancer therapy resistance (Crosby et al., 2009; Fasanaro et al., 2009; Shen et al., 2013). Other studies that use the microarray method reveal that in a hypoxic environment, different miRNAs are expressed (hypoxia- regulated miRNAs; HRMs). Some miRNAs, such as miR-210, -155, -372/373, and -10b, are up-regulated in hypoxia (Crosby et al., 2009; Neal et al., 2010; Loayza-Puch et al., 2010; Bruning et al., 2011); in contrast, miR-20b and miR-200b were down-regulated in hypoxic conditions (Chan et al., 2011). The α and β subunits of HIF-1 may adjoin both ends of the HRE in the promoter regions of HRM genes. In hypoxia, the affinity between the HIF-1 subunits and HRE can intensify, causing the transcription of HRMs to increase. HIF-1 regulates the expression of a number of HRMs, including miR-210, -155, and -373, by modulating HREs (Huang et al., 2009; Bruning et al., 2011). It has recently been discovered that in hypoxic conditions, miR-210 regulates mitochondrial metabolism. A number of researchers have shown that the function of miR-210 is to inhibit parts of the mitochondrial electron transport chain (ETC) complexes stages, causing mitochondria to switch from oxidative phosphorylation to glycolysis (Fasanaro et al., 2009; Huang et al., 2009; Chen et al., 2010; Favaro et al., 2010). miR-210 targets the iron-sulfur cluster scaffold homolog and cytochrome c oxidase assembly factor (COX10) proteins, inhibiting mitochondrial respiration. Other important cell metabolism structures that are the target of miR-210 include GPD1-L (Favaro et al., 2010), NADH dehydrogenase (ubiquinone) 1 alpha sub-complex 4 (NDUFA4) (Giannakakis et al., 2008), and succinate dehydrogenase complex (Puissegur et al., 2011).

## HIF-1-Induced Metabolic Adaptations

**FIGURE 4:**
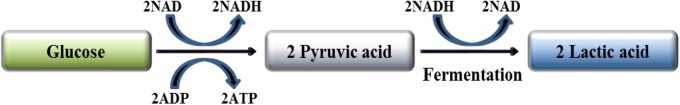
Anaerobic glycolysis. Glycolysis provides two ATP molecules and does not require oxygen. One glucose molecule is converted into two pyruvate molecules, which are subsequently fermented to two lactic acid molecules.

Metabolic alterations were among the first biochemical distinctive feature of cancer cells to be discovered and increased understanding of tumor metabolism enables the elucidation of novel targets and informs the development of new anticancer therapies (Koukourakis et al., 2006; Giatromanolaki et al., 2017). The difference between normal tissue and cancer metabolism was first noted in the 1920s; with healthy cells, in normoxic conditions, glucose is broken down into pyruvate, which is then further catabolised via the tricarboxylic acid cycle and oxidative phosphorylation in the mitochondria. In normal cells, the rate of glycolysis is suppressed by the presence of oxygen (Pasteur effect). Mitochondrial activity maintains elevated levels of ATP, this leads to allosteric inhibition of the enzyme responsible for glycolysis, phosphofructokinase (PFK) (Alfarouk et al., 2014). In hypoxic conditions the end product of anaerobic glycolysis is pyruvate that is then metabolized to lactate. Tumors, in contrast to non-malignant tissue, tend to rely heavily on an increased rate of glycolysis to support their energy demands even when oxygen is plentiful, a phenomenon termed aerobic glycolysis or the Warburg Effect (Liberti and Locasale, 2016) (Figure 4).

Warburg (1930) noticed that, compared to normal cells, tumor cells exhibited higher glucose metabolism rates and preferentially utilized glycolysis over oxidative phosphorylation, even when oxygen levels were adequate (House et al., 1956; Warburg, 1956). Since then, a wide range of types of tumors have been found to manifest aerobic glycolysis and evidence has been accumulated that cancer progression is accompanied by an overall restructuring of metabolism (Fritz and Fajas, 2010).

The influence of HIF-1α on glycolytic metabolism is well established (Maxwell et al., 2001); glycolysis in tumors could possibly be driven by HIF-1α stabilization, independently of the hypoxic environment. According to an earlier survey of cancer cell lines, there was about a 50% incidence of HIF-1α stabilizing under normoxic conditions in cancers (Welsh et al., 2004; Robey et al., 2005). Further to this the enzymes responsible for the shift in metabolism by the repression of oxidative phosphorylation and promotion of anaerobic glycolysis are controlled by HIF-1α (Weidemann and Johnson, 2008; Danquah et al., 2011). HIF-1α initiates over-expression in tumor cells and raises the activity of a number of glycolytic protein isoforms which are different to those present in non-malignant cells. Such isoforms include: adenylate kinase-3; aldolase-A,C (ALDA,C); carbonic anhydrase-9; enolase-1 (ENO1); glucose transporter-1,3 (Glut-1,3); glyceraldehyde phosphate dehydrogenase (GAPDH); hexokinase 1,2 (HK1,2); lactate dehydrogenase-A (LDHA); phosphofructokinase L (PFKL); phosphoglycerate kinase 1 (PGK1); and 6-phosphofructo-2-kinase/gructose-2,6-bisphosphate-3 (PFKFB3); (Ke and Costa, 2006; Semenza, 2010).

Hypoxia-inducible factor directly up-regulate the genes coding for glucose transporters such as Glut-1 and the enzymes of the glycolytic pathway. HIF-1α induces the expression of pyruvate dehydrogenase kinase 1 (PDK1), which phosphorylates and consequently inhibits pyruvate dehydrogenase (PDH) (Kim et al., 2006), which converts pyruvate to acetyl-CoA. Reduced PDH activity under hypoxic conditions decreases the entry of acetyl-CoA into the Krebs cycle, reducing the amount of substrate available for downstream mitochondrial respiration and as a result, oxygen consumption. Additionally, HIF-1α enables cells to regulate to the reduced intracellular pH that occurs as a consequence of increased anaerobic glycolysis and the resulting lactic acid production (Weidemann and Johnson, 2008).

In a previous analysis of RT-qPCR data, the mRNA expression of the HK-1 and 2, PKM-2, LDH-A, and Glut-1 genes, has demonstrated cross tissue variation in expression and correlations with clinical or pathological characteristics. Degrees of expression of Glut-1, LDH-A, HK-1, PKM-2 and VDAC-1 mRNA were observed to be significantly greater in primary tumor/liver metastasis tissues relative to normal tissue mucosa (Graziano et al., 2017).

A significant contributing mechanism responsible for this aberrant behavior is believed to be mitochondrial dysfunction (Chandra and Singh, 2011; Owens et al., 2011). Mitochondria are highly dynamic organelles. Mitochondrial morphology is regulated by fission/fusion mechanisms, which are modulated according to changes in oxygen availability. Under hypoxia, faster glucose consumption occurs in an attempt to maintain ATP production using less efficient anaerobic glycolysis (Lum et al., 2007) and a shortage in the provision of substrates like acetyl-CoA and O_2_ to mitochondria induces major structural, functional, and dynamical changes. The structural and dynamical changes are characterized by impairment of fusion process that leads to mitochondrial depolarization, loss of mitochondrial DNA (mtDNA) that may be associated with altered respiration rates, and uneven distribution of the mitochondria within cells (Jezek and Plecitá-Hlavatá, 2009). Under continuous hypoxia, neurons have reduced mitochondrial size and altered mitochondrial morphology, possibly in accordance with changes in the activity of nitric oxide synthase (Guo et al., 2008). As described by López-Ríos et al. (2007) and Moreno-Sánchez et al. (2007), many alterations in mitochondria expression and function are associated with the increased rate of glycolysis in rapidly expanding tumors. In addition to a decrease in the number of mitochondria within the cells, there is an associated decrease in expressed levels of oxidative enzymes and transporters, and conversely, an increase in a protein (IF1) which inhibits mitochondrial ATP synthase. Furthermore, activation of glycolysis results in lower levels of oxidative phosphorylation (López-Ríos et al., 2007; Moreno-Sánchez et al., 2007). The increase in the rate of glucose metabolism through aerobic glycolysis is significant, with the rate of lactate production from glucose being between 10 and 100 times greater than total oxidation of glucose in mitochondria. However, the quantity of ATP synthesized in a given period of time is similar regardless of the form of glucose metabolism used (Shestov et al., 2014; Liberti and Locasale, 2016). Several factors contributing to the switch to aerobic glycolysis in various cancer types were identified by Zheng (2012) and included oncogene activation, tumor suppressor loss, the hypoxic microenvironment, mutations in mtDNA and the tissue of origin (Zheng, 2012).

Aerobic glycolysis exhibits a number of essential, but poorly understood, benefits which facilitate the preference for glycolysis rather than mitochondrial oxidation in cancer cells, despite the stoichiometric indication that mitochondrial oxidation is required to maintain high glycolysis rates (De Souza et al., 2011). Production of vital secondary metabolites for generation of lipids, proteins and nucleic acids to promote cancer cell development and proliferation is a key role for aerobic glycolysis (Graziano et al., 2017). Tumors may benefit from the cell-independent effects of lactate production, as lactate may interfere with normal tissue structure, thus making tumor cells more invasive (Locasale and Cantley, 2010). While a correlation between rates of glycolysis and tumor aggressiveness has been demonstrated *in vitro*, not all tumor cell types depend on glycolysis for ATP generation. Some tumor types utilize both glycolysis and oxidative phosphorylation, or some completely on oxidative phosphorylation for ATP supply (Moreno-Sánchez et al., 2007; Zheng, 2012). The evidence of this correlation is demonstrated in comparing the non-invasive MCF7 breast cancer cell line (non-invasive), which has much lower glucose consumption with the more aggressive and highly invasive MDA-MD-231 breast tumor cell line (Gatenby and Gillies, 2004). The conversion of glucose into pyruvate and then lactate leads to a lowering of intracellular pH, which is countered by the export of lactate from the cell, causing extracellular acidification and consequently suppression of the anticancer immune response (Locasale and Cantley, 2010). Tumor- associated fibroblasts express the monocarboxylate co-transporters MCT1 and MCT4, which transport important monocarboxylates such as lactate and pyruvate (Koukourakis et al., 2006). Lactate can be sequestered by stromal cells through the monocarboxylate transporters to regenerate pyruvate that either can be used for oxidative phosphorylation or can be used to refuel the cancer cell through the lactic acid cycle (Koukourakis et al., 2006).

Successful treatment approaches have so far not been accomplished, despite endeavors to use compounds like 2-deoxyglucose to inhibit aerobic glycolysis in cancer cells. Suppression of lactate dehydrogenase and the blocking of monocarboxylate transporters carrying lactate over the plasma membrane are among the novel treatment strategies, with multiple targets in the glycolytic process that are presently being assessed (Fantin et al., 2006; Le et al., 2010). There are a number of methods of disrupting glycolysis; developing glycolytic inhibitors capable of acting with very high specificity is a target for the pharmaceutical industry that may translate into a clinical success (Ganapathy-Kanniappan and Geschwind, 2013; Ganapathy-Kanniappan et al., 2013).

## Glucose Transporters (Gluts)

Glycolysis produces only two molecules of ATP from a single molecule of glucose, compared with oxidative phosphorylation, which produces 36 ATP molecules; cancer cells therefore demand high fluxes of glucose to make up for this shortfall and satisfy their energy demand (Alberts et al., 2008). Almost all mammalian cells utilize glucose as a major source for energy and ATP generation. Glucose is also a vital substrate for the synthesis of triglycerides, glycoproteins and glycogen (Martinez-Outschoorn et al., 2017). Tissue specific glucose transport is conducted *via* the 13 facilitative glucose transporter family (Glut/SLC2A) members, which vary by their mode of regulation and affinities, for example, Glut-5 transports fructose (Palm and Thompson, 2017). This process enables the facilitative glucose transporters to maintain the glucose concentration gradient, an energy-independent process, ensuring a continuous source of glucose for metabolism (Zhou et al., 2017). Gluts are overexpressed in solid tumors and are directly linked to a poor prognosis in cancer patients. The Glut-1 isoform is overexpressed in a variety of malignant neoplasms, including brain, hepatic, and pancreatic tumors (Shibuya et al., 2015; Barron et al., 2016) and correlates with tumor aggressiveness and poor prognosis (Kunkel et al., 2003). Glut-1 overexpression also correlates with tumor grade and distance from stromal blood supply, which suggests a link with tumor hypoxia (Mendez et al., 2002). In support of this hypothesis it has been found to be abundantly expressed in hypoxic regions surrounding necrotic foci of breast and colorectal cancers (Chen et al., 2017). Prolonged hypoxia induces the expression of Glut-1 *via* the inhibition of oxidative phosphorylation and upregulation of HIF-1 (Giatromanolaki et al., 2017).

Researchers including Suh and Han (2013) and Ancey et al. (2018) have shown that Glut-1 synthesis and transfer to the cell membrane are positively influenced by multiple hypoxia-related components, including VEGF receptor and calcium channel transactivation (Suh and Han, 2013; Ancey et al., 2018). Recently, the same researchers (Ancey et al., 2018) have demonstrated the actions of Glut-1 and Glut-3 in enhancing cancer cell metabolism. Simpson et al. (2008) demonstrated that, as glucose transporters, Glut-1 and Glut-3 expression is up regulated to increase glucose uptake and ultimately increase HIF-1 driven glycolysis. Furthermore, earlier work in an erythroleukemia mouse model demonstrating that Glut-1 expression is positively related to resistance to multiple anti-cancer therapies, including vincristine (Trendowski et al., 2015). Both Glut-1 and Glut-3 are overexpressed in human tumors, with Glut-1 expressed with greater frequency than Gut-3. However, the Glut-3 type is the major glucose transporter expressed in human brain tumors of glial origin (Simpson et al., 2008). Subsequently, other researchers demonstrated a positive relationship in HIF-1α and Glut-3 expression in human glioma tumors, by immunohistochemistry and western blotting (Liu et al., 2009). These markers, HIF-1α and Glut-3, show a positive correlation with the grade of glioma and there is evidence to support a relationship between these markers and tumor formation and rate of progression. On this basis, we can conclude that the presence and level of these markers are robust and valid reporters for glioma development and growth (Liu et al., 2009).

## The Role of Hypoxia in Resistance to Conventional Chemo- and Radiation Therapy

There is a significant body of evidence that demonstrates hypoxia-induced therapeutic resistance (Trédan et al., 2007; Khawar et al., 2015). In part, this association may be due to the fact that many anticancer drugs are large molecules, which are not able to diffuse to target tissue due to the poorly formed vasculature. Moreover, hypoxic tumor cells are distant from the blood supply and as a result only a proportion of them may be exposed to a lethal dose of a cytotoxic agent (Trédan et al., 2007).

Tumor-associated hypoxia is shown to promote drug resistance, specifically through increased expression and amplification of the genes for the *P*-glycoprotein (P-gp) membrane exporter. The consequence of increased expression of P-gp is a reduction in cellular sequestration of multiple anti-cancer drugs, and it is likely that this pathway is correlated with tumor resistance to topoisomerase II-targeted drugs (Trédan et al., 2007). Radioresistance is the negative effect of tumor hypoxia that impacts the ability of radiotherapy to treat tumors (Harrison et al., 2002; Moeller et al., 2007). Tumor sensitivity to radiation exposure rapidly declines when the local pO_2_ is less than 25–30 mmHg (3.3–3.9%) (Ruan et al., 2009). Oxygen enhancement is the term used to describe the process by which hypoxia confers radioresistance within a tumor. As a consequence of radiotherapy, via ionization or associated with oxygen-favoring radicals, such as hydroxyl radical and superoxide, resulting from ionization of water surrounding DNA, tumor cell DNA is damaged. In short, radiotherapy results in DNA breakages, and failure by the cell to repair the damage frequently results in cell death. In hypoxia situations, the cells have increased ability to conduct repairs to the disrupted DNA, while in the presence of oxygen; stable peroxides are created between oxygen and the free DNA ends, which are substantially more resistant to cellular repair (Hu and Harrison, 2005; Hall and Giaccia, 2006; Ruan et al., 2009). Resistant tumor cells can remain viable after treatment, which leads to resistant subpopulations of cells and poor local control for the patient (Moeller et al., 2007). In addition, there is an association between HIF-1, the tumor blood vessel network, and radiation resistance (Moeller et al., 2004), inasmuch as HIF-1 activity is influenced by radiation. The activity of HIF-1 in a tumor can nearly double 24–28 h after exposure to radiation. This phenomenon is restricted to *in vivo* tests only, as it relies on essential input from the tumor microenvironment (Moeller et al., 2007). Following radiotherapy, tumors may undergo a transient rise in oxygen tension, or reoxygenation, due to the reduction in diffusion-limited (chronic) hypoxia as a consequence of cytoreduction (Bayer and Vaupel, 2012). Subsequently, radiation-induced re-oxygenation of hypoxic tumor cells leads to the production of ROS that induce HIF-1, which, in turn, activates the expression of cytokines, including VEGF and basic fibroblast growth factor (bFGF), which confer radio-protective effects on neighboring endothelial cells. Ultimately, this leads to inhibition of endothelial apoptosis via induction of anti-apoptotic signals to tumor blood vessels, promoting an additional mechanism of radiation resistance (Moeller et al., 2007; Zhou et al., 2017).

## Conclusion

Tumor cells have three major specifications, namely, heightened energy generation, adequate macromolecular biosynthesis and redox balance preservation, which must be balanced by the metabolic adaptations. These processes must be closely investigated to identify the weakness in tumor metabolic pathways and thus formulate effective treatment strategies. Creation of therapeutic approaches capable of retarding tumor progression, enhancing treatment response and achieving favorable clinical results is the overarching aim. Furthermore, an enhanced appreciation of tumor metabolism, specifically the relationship between aerobic glycolysis and adaptive malignant growth, may direct the advancement of tumor-targeting and normal tissue-sparing neo-therapeutics. Driving permissive cellular and tissue survival factors, hypoxia-led metabolic change in cancers permits enhanced malignancy, and subsequently results in lower survival rates via enhanced therapy resistance and augmented metastatic capacity. Clarifying the processes by which hypoxia drives metabolic change at the cellular level will facilitate a strategic focus on the defined pathways, ultimately ensuring death of the tumor cells.

## Author Contributions

WAT, TD, RA-J, and NF contributed to the planning and writing and editing of this review article.

## Conflict of Interest Statement

The authors declare that the research was conducted in the absence of any commercial or financial relationships that could be construed as a potential conflict of interest.
